# Improving Abbreviated Mental Test Score Documentation in Acute Medical Admissions Using a Systems Engineering Initiative for Patient Safety

**DOI:** 10.7759/cureus.119527

**Published:** 2026-11-12

**Authors:** Kiransoy J Koikara, Betsy Babu

**Affiliations:** 1 Accident and Emergency, Queen Elizabeth Hospital King's Lynn NHS Foundation Trust, Cambridge, GBR; 2 Internal Medicine, Medical University of Sofia, Sofia, BGR

**Keywords:** abbreviated mental test score (amts), acute medicine, cognitive screening tools, patient health safety, quality improvement project (qip), seips

## Abstract

Introduction

Reliable documentation of cognitive screening is important in acute medical care because an absent result does not show whether screening was omitted, deferred, or completed but not recorded. This quality improvement project aimed to improve documentation of the Abbreviated Mental Test Score (AMTS) in acute medical admissions and to explore staff-reported barriers using the Systems Engineering Initiative for Patient Safety (SEIPS) framework.

Methods

This was a single-centre, two-cycle quality improvement project at an NHS hospital in England. The supplied project record describes an initial audit in March and a re-audit in June. The primary process measure was the proportion of audited records containing a completed AMTS form. Staff feedback was reviewed descriptively and mapped to person, task/technology, organisational, and environmental factors. A multicomponent improvement package included teaching, posters, awareness activities, and discussion at a grand round. The stated local target was 60% completion.

Results

At baseline, 28 of 60 records contained a completed AMTS form (46.7%). At re-audit, 40 of 58 records contained a completed form (69.0%), an absolute increase of 22.3 percentage points. The re-audit was 9.0 percentage points above the local 60% target. Between 00:00 and 07:59, completion increased from 2/11 (18.2%) to 4/10 (40.0%). Staff feedback identified competing clinical demands, fatigue, forgetting, uncertainty about expectations, reduced overnight staffing or supervision, and patients who were asleep or unable to engage.

Conclusion

Recorded AMTS completion was higher at re-audit after a package of education and awareness measures. The uncontrolled before-and-after design, modest sample, and incomplete documentation of implementation and governance limit causal interpretation. The findings support further local testing of workflow-linked prompts, clear task ownership, and a documented approach to clinically appropriate deferral.

## Introduction

Clinical and service context

Acute illness in older adults may coexist with cognitive impairment or delirium, and changes in attention, awareness, or cognition can be missed when assessment is difficult to distinguish from the patient’s baseline. Delirium is associated with important adverse consequences, and hypoactive presentations may be subtle; national guidance therefore emphasises vigilance, documentation of indicators, and appropriate assessment when delirium is suspected [1,2]. A brief cognitive screen can contribute to assessment, but it is not a standalone diagnosis and should be interpreted alongside the clinical history, collateral information, examination, communication needs, sensory impairment, and the patient’s usual cognitive function.

AMTS: original source and appropriate interpretation

The Abbreviated Mental Test Score (AMTS) was described by Hodkinson in 1972 as a shortened 10-question version of a longer mental test, developed to assess mental impairment in older people [1]. Later work has compared the original AMTS with variants and highlighted that versions of the tool should not be assumed to be interchangeable without attention to their content and validation [2]. In this project, the outcome was completion of the locally used AMTS form; the audit did not evaluate diagnostic accuracy, inter-rater reliability, or patient outcomes. Accordingly, an increase in documented completion should not be interpreted as evidence that cognitive impairment or delirium was detected more accurately.

The current National Institute for Health and Care Excellence (NICE) delirium guideline recommends assessment with the 4AT when indicators of delirium are identified in most hospital settings, with the Confusion Assessment Method for the Intensive Care Unit (CAM-ICU) or Intensive Care Delirium Screening Checklist (ICDSC) in critical care and post-operative recovery settings [1]. The local clinical rationale for requiring AMTS, the population to whom it applies, and its relationship to the hospital’s delirium pathway should therefore be confirmed by the responsible clinical team. This manuscript evaluates a local documentation process and does not claim that AMTS is the preferred or sufficient tool for diagnosing delirium.

Documentation reliability as a systems problem

A blank field is ambiguous: it may represent an omitted task, a test deferred because the patient was asleep or unable to participate, an assessment completed but not entered, or a clinical decision that the task was not appropriate at that time. These possibilities have different implications. A reliable process should support completion when appropriate and make deferral and follow-up visible, rather than incentivising completion regardless of clinical circumstances.

The original Systems Engineering Initiative for Patient Safety (SEIPS) model describes how people, tasks, tools and technologies, the physical environment, and organisational conditions interact to shape healthcare work. SEIPS 2.0 extends this systems perspective by considering how work-system configurations and engagement change over time [3]. This framework was selected because staff may understand the purpose of screening yet still miss documentation when faced with interruptions, competing admissions, unclear task ownership, or a workflow prompt that is easy to overlook. A systems analysis can therefore identify changes beyond individual reminders or education.

Quality improvement rationale and aims

Quality improvement reporting benefits from a clear description of the local problem, available knowledge, rationale for the intervention, context, measurement, and learning. Standards for QUality Improvement Reporting Excellence (SQUIRE) 2.0 provides a reporting framework for systematic efforts to improve healthcare [4]. The Model for Improvement and Plan-Do-Study-Act (PDSA) cycles provide a practical approach to testing changes iteratively, although two aggregate audit points alone cannot establish sustained change.

The project had four aims: (1) measure AMTS documentation completion across two audit cycles and by time of day; (2) review staff feedback about barriers, with particular attention to overnight work; (3) introduce education and awareness measures informed by a SEIPS analysis; and (4) compare the completion proportion at re-audit with baseline and the local 60% target.

## Materials and methods

Design and setting

This was a single-centre, two-cycle quality improvement project (QIP) conducted at an NHS hospital in England. The baseline audit was conducted in March and the re-audit in June 2026. The project evaluated completion of the locally used AMTS documentation form in acute medical admissions. The specific clinical area, eligibility criteria, sampling method, and confirmation that identical inclusion criteria were applied in both cycles should be verified by the project team before submission. The project was framed as a local quality improvement initiative and reported with reference to the SQUIRE 2.0 guidelines for transparent reporting of healthcare improvement work [4].

Measures and data collection

The primary process measure was the proportion of audited records containing a completed AMTS form, calculated by dividing the number of records with a completed form by the total number of records audited in each cycle. The AMTS is a brief cognitive screening instrument originally described by Hodkinson for assessing mental impairment in older people [1]. Later research has compared the original AMTS with variants used to screen for cognitive impairment, highlighting the importance of identifying which version is used [2]. In this project, the outcome was completion of the locally used AMTS form; the audit did not assess diagnostic accuracy, inter-rater reliability, or patient outcomes. The baseline audit included 60 records, of which 28 had a completed AMTS form. The re-audit included 58 records, of which 40 had a completed form. The local target was 60% completion; this was a locally agreed process-improvement target, not an evidence-based clinical threshold.

Time-of-day stratification

Completion was also examined by time of day, with particular attention to the overnight period (00:00-07:59). This stratification was intended to identify potential differences in documentation patterns across the 24-hour admission period. The overnight subgroup comprised 11 records in cycle 1, with two completed forms, and 10 records in cycle 2, with four completed forms. The denominators are small, so subgroup percentages were interpreted descriptively.

Staff feedback and barrier assessment

Staff survey responses and night-shift poll and feedback materials were reviewed descriptively to identify perceived barriers to AMTS documentation and suggestions for improvement. The available project materials did not include the complete questionnaire, survey distribution method, number of staff invited, response rate, respondent characteristics, item-level response counts, or a complete dataset of verbatim responses. Accordingly, the findings are presented as themes identified in the project materials rather than as estimates of the prevalence of particular barriers among staff.

SEIPS-informed intervention

The SEIPS framework was used to organise the identified barriers and inform the selection of improvement measures. SEIPS describes how people, tasks, tools and technologies, the physical environment, and organisational conditions interact to shape healthcare work [3]. Person-related factors identified in the project materials included knowledge, fatigue, forgetting, and patients’ alertness or ability to participate. Task and technology factors included competing clinical priorities, workload, and the passive prompt within the clerking proforma. Organisational and environmental factors included reduced overnight staffing, limited supervision, unclear accountability, interruptions, and admission pressures. The intervention package comprised teaching, posters, awareness activities, and discussion at a grand round. Staff suggestions included induction teaching, visual reminders, nursing prompts, consultant emphasis during post-take ward rounds, and additional training in delirium assessment. These components were considered collectively; the project design did not permit the independent effect of individual components to be determined.

Analysis

The analysis was descriptive. AMTS documentation completion was summarised using counts and percentages for each audit cycle. The absolute change was calculated by subtracting the baseline completion percentage from the re-audit completion percentage and was expressed in percentage points. Overall completion increased from 28/60 (46.7%) at baseline to 40/58 (69.0%) at re-audit. Overnight completion increased from 2/11 (18.2%) to 4/10 (40.0%). No inferential statistical testing was reported. The Model for Improvement and PDSA cycles provide a practical framework for iterative testing of changes; however, two aggregate audit points alone cannot establish sustained change or demonstrate that the intervention caused the observed difference.

## Results

Overall completion

In the first audit cycle, 28 of 60 audited records contained a completed AMTS form, corresponding to a completion rate of 46.7%. The remaining 32 records (53.3%) had no completed AMTS form recorded. In the second cycle, 40 of 58 records contained a completed form (69.0%), while 18 records (31.0%) did not. Across the two audit samples, the recorded completion proportion therefore increased by 22.3 percentage points. The denominators differed slightly between cycles (60 records at baseline and 58 at re-audit), so the comparison is between the proportions observed in each sample rather than a paired comparison of the same patients (Table 1).

**Table 1:** Abbreviated Mental Test Score (AMTS) documentation completion across the two audit cycles, including overall and overnight results. Percentages are calculated using the number of records audited in each subgroup. pp, percentage points

Audit cycle/subgroup	Records audited, n	AMTS completed, n (%)	AMTS not completed, n (%)	Change in completion (percentage points)	Comparison with 60% target	Interpretation
Overall audit results						
Cycle 1	60	28 (46.7%)	32 (53.3%)	Reference cycle	13.3 pp below target	Fewer than half of audited records documented completion.
Cycle 2	58	40 (69.0%)	18 (31.0%)	+22.3 pp vs. cycle 1	9.0 pp above target	Completion was higher at re-audit.
Over-cycle change	-	+12 completed records	-14 incomplete records	+22.3 pp	Target exceeded in cycle 2	The proportions changed between two audit periods.
Overnight subgroup (00:00-07:59)						
Cycle 1	11	2 (18.2%)	9 (81.8%)	Reference cycle	Not separately specified	Completion was lower overnight than in the overall cycle 1 sample.
Cycle 2	10	4 (40.0%)	6 (60.0%)	+21.8 pp vs. cycle 1	Not separately specified	Completion increased but remained below 50.
Over-cycle change	-	+2 completed records	-3 incomplete records	+21.8 pp	Not separately assessed	Small subgroup denominators mean each record has a substantial effect on the percentage.
Local improvement target						
Target for overall completion	-	60% completion	40% not completed at target	-	Locally agreed threshold	This was a local process target.

Performance against the local target

The project target was 60% completion. Baseline performance was 13.3 percentage points below this target, whereas the re-audit result was 9.0 percentage points above it. Despite reaching the target in the second cycle, 18 of 58 records (31.0%) still had no completed AMTS form recorded. The result therefore represents improvement against the project’s process measure, rather than a fully reliable documentation process. The 60% threshold was locally agreed for this project and should be interpreted as a local improvement benchmark, not as an evidence-based clinical standard.

Time-of-day pattern

During the overnight interval, defined in the project materials as 00:00-07:59, two of 11 records had a completed AMTS form in cycle 1 (18.2%). In cycle 2, four of 10 records had a completed form (40.0%), an absolute increase of 21.8 percentage points. Conversely, nine of 11 overnight records in cycle 1 (81.8%) and six of 10 in cycle 2 (60.0%) had no completed form recorded. The overnight completion proportion was therefore higher at re-audit but remained below 50%. The subgroup denominators were small, meaning that one or two records could materially alter the percentages; the difference should consequently be interpreted cautiously. The available data also do not establish whether an absent form represented an omitted assessment, a clinically appropriate deferral, or an assessment completed but not documented.

The supplied charts also suggested that incomplete documentation was not confined to the overnight interval: in several later evening intervals, the number of completed forms appeared lower than the number of patients recorded. However, the source materials available for this manuscript did not provide a complete set of exact counts for each time interval. No additional interval-specific percentages have therefore been calculated. A verified time-of-day dataset would be needed to determine whether completion differed consistently across day, evening, and night periods.

Staff feedback and SEIPS analysis

The staff survey, night-shift poll, and presentation materials described several potentially interacting barriers to AMTS documentation. Workload-related themes included lack of time, competing clinical priorities, interruptions, and higher workload or reduced staffing overnight. Person-related themes included forgetting and fatigue, while organisational themes included limited supervision or accountability and uncertainty about local protocols, training, or expectations. Patient-related circumstances were also described: patients could be asleep, less alert, agitated, or otherwise unable to engage with the assessment at the time of admission. These themes indicate that a blank field may arise in different circumstances and should not automatically be interpreted as the same type of omission.

Suggestions recorded in the project materials included teaching during staff induction, posters and visual reminders in clinical areas, nursing prompts, emphasis from consultants during post-take ward rounds, and further training in delirium assessment. The project team organised the reported barriers using the SEIPS domains of person, task/technology, organisation, and environment. Teaching, posters, awareness activities, and discussion at a grand round formed the reported multicomponent response. The available materials do not quantify staff participation or the reach of each intervention, and the components were introduced as a package; their individual contributions to the change in completion cannot be estimated.

Figure 1 presents an overview of the two-cycle AMTS QIP and the SEIPS-informed barrier domains.

**Figure 1:**
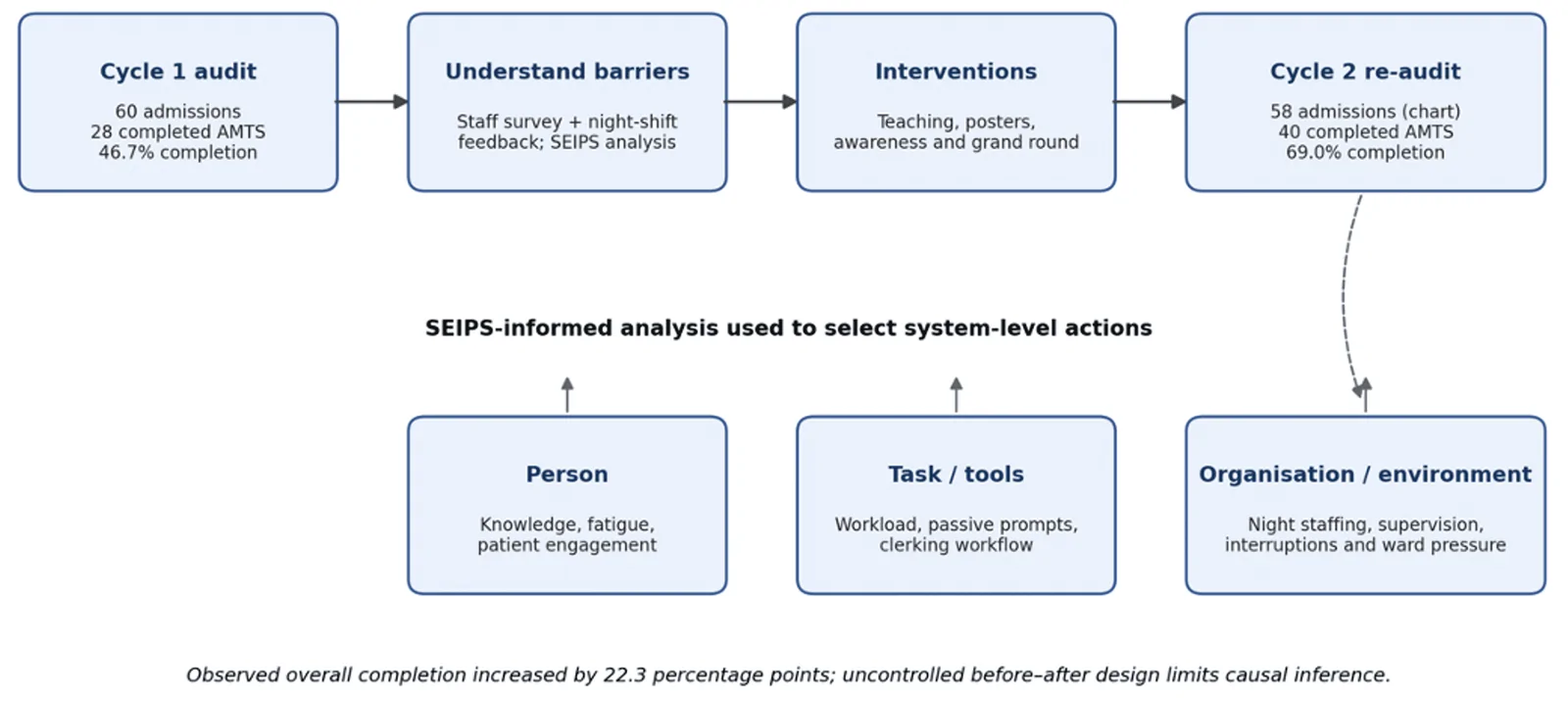
Overview of the two-cycle Abbreviated Mental Test Score (AMTS) quality improvement project and the Systems Engineering Initiative for Patient Safety (SEIPS)-informed barrier domains.

## Discussion

At an NHS hospital in England, recorded AMTS completion increased from 46.7% in the March audit to 69.0% in June after the team introduced teaching, posters, awareness activities, and discussion at a grand round. The difference between cycles was 22.3 percentage points, and the June result was above the 60% target. Overnight completion also increased, from 18.2% to 40.0%, but remained low enough to warrant further work. These findings suggest that the context of care, particularly overnight, should remain part of the improvement strategy.

A useful feature of this project was the decision to consider several possible causes of missed documentation rather than treating it only as an individual knowledge gap. Feedback described challenges involving clinician workload and fatigue, patient readiness, the clerking task itself, reminders, staffing, and supervision. A prompt that is easy to overlook may not be enough when staff are managing urgent clinical work. Teaching and posters can make expectations more visible, but further cycles could examine whether clearer task ownership, a workflow-linked prompt, and a documented plan for patients who cannot participate at that time improve reliability. These options should be developed with the clinical team and checked for unintended effects on workload.

The project can be reported with reference to SQUIRE 2.0, which was developed to improve the transparency of healthcare quality improvement reports [4]. In particular, readers need to understand the local context, what was changed, how outcomes were measured, and what was learned during implementation. The change observed between two audits is encouraging, but this design cannot show that the intervention package alone produced the improvement.

This project has limitations. It was carried out at one centre and did not include a concurrent comparison group, so changes in staffing, workload, patient characteristics, or local practice may have contributed to the difference between cycles. The second-cycle counts were confirmed from the project chart rather than a raw dataset supplied for independent checking. The materials available for this draft do not provide the full survey denominator and response distribution, exact cycle dates and year, intervention reach, or evidence that the improvement was sustained. In addition, a completed form does not establish that the assessment was accurate, clinically appropriate, or associated with better patient outcomes.

The next cycle should use a verified dataset and the same eligibility rules in both periods. The team could set out a time-specific target, review completion by shift, and record reasons for non-completion, including when a patient is asleep or temporarily unable to engage. A time-ordered display of results may help distinguish a sustained change from normal variation. The team could also consider balancing measures, such as the time required to complete the assessment, and repeat measurement after staff induction or rotation to assess whether gains persist.

Rationale for the improvement approach

Why Documentation Reliability Matters

A documented screening result creates a record that the assessment was considered and allows subsequent clinicians to understand what was found at the time of admission. In a busy acute medical environment, a blank field is ambiguous: it may mean that the assessment was overlooked, that the patient could not participate, that the clinician judged it inappropriate at that moment, or that the assessment occurred but was not recorded. These possibilities have different implications for improvement. A reliable process should therefore aim not only to increase completed forms but also to make the reason for non-completion visible and clinically meaningful. The present project measured documentation completion, not diagnostic accuracy or patient outcomes; the distinction is important when interpreting the results.

AMTS in the Context of Cognitive Assessment

The AMTS is a brief screening instrument and should be interpreted in the context of the clinical presentation, communication needs, sensory impairment, language, education, and the patient’s usual cognitive function. A screening score is not equivalent to a diagnosis of dementia or delirium. NICE guidance recommends assessing risk factors for delirium and using a validated assessment when delirium is suspected; its current recommendations identify the 4AT for assessment in most hospital settings, with other tools in critical care and recovery settings [5]. Accordingly, this QIP should be understood as an intervention to improve completion of a locally used AMTS process, not as evidence that AMTS alone is the preferred or sufficient tool for diagnosing delirium. The local rationale for using AMTS, including the population and pathway in which it is required, should be confirmed by the clinical team before submission.

Quality Improvement Theory and Iterative Testing

Quality improvement differs from a one-off audit because the purpose is to use measurement and learning to change a local process. The Model for Improvement asks teams to define an aim, select measures, and test changes through PDSA cycles [6]. In this project, the first audit provided a baseline, staff feedback helped explain the pattern, a package of awareness and teaching interventions was introduced, and a second audit assessed whether completion had changed. The two-cycle structure is appropriate for an initial local improvement effort, but a longer sequence of measurements would be needed to understand whether the observed difference persisted and whether it reflected a sustained process change rather than short-term attention following teaching.

Why a Systems Approach Was Selected

A systems approach is useful when staff understand that a task matters but completion is inconsistent. SEIPS frames performance as the result of interactions between people, tasks, tools and technology, the physical environment, and organisational conditions [3,7]. Applied to this project, a staff member may know how to complete AMTS but still omit it because of competing admissions, interruptions, a patient who is asleep, uncertainty about who owns the task, or a form that does not prompt action at the right time. Treating these situations solely as failures of individual diligence would risk choosing interventions that do not address the conditions producing the problem. The SEIPS analysis therefore supported a combination of education, visual reminders, team discussion, and consideration of workflow and supervision.

Intervention components and proposed mechanisms

Teaching and Presentation

Teaching was intended to reinforce the purpose of AMTS documentation and clarify expectations for clinicians involved in acute medical clerking. Presentation at a grand round also created an opportunity to discuss the audit findings with a wider clinical audience and collect suggestions. Education can improve shared understanding, but its effect may be limited if the task competes with urgent clinical responsibilities or if new staff do not receive the same message. The materials supplied do not quantify attendance, staff reach, or knowledge before and after teaching; therefore, the manuscript does not claim a measured educational effect.

Posters and Awareness Prompts

Posters and awareness activities were intended to make the expected process more visible at the point of work. Their potential benefit depends on placement, readability, and whether they are seen during the clerking workflow. Visual reminders can lose impact when staff become familiar with them or when the clinical environment is crowded with notices. Future testing could compare different locations and wording, while asking staff whether the prompt was noticed and whether it helped at the time the task was due. The project data do not isolate the effect of posters from the other components, so the overall change should be attributed to the package as a whole rather than to any single intervention.

Grand-Round Feedback and Shared Ownership

The grand-round discussion generated suggestions that were considered as part of the improvement work. This is relevant because a process that relies on one professional group may fail when responsibilities cross roles or shift handovers. A future version of the local process could explicitly define who completes AMTS, when it should be done, how completion is recorded, and what to do when the patient is temporarily unable to participate. Such expectations should be agreed locally rather than assumed from the present audit. Feedback from staff who work overnight is particularly important because the initial findings indicated a lower completion proportion during the overnight period.

Interpretation of the results

Overall Change Relative to the Target

The proportion of completed forms rose from 46.7% in March to 69.0% in June. The absolute change was 22.3 percentage points. Relative to the baseline proportion, this corresponds to an increase of approximately 47.7%; however, absolute percentage-point change is the more straightforward measure for communicating progress against a target. The June proportion was 9.0 percentage points above the stated 60% target. This target was a process threshold for the QIP and should not be interpreted as evidence that 60% is an evidence-based clinical standard. A target of 60% also means that nearly one-third of recorded patients still lacked a completed form in the second cycle, so the result represents progress rather than a fully reliable process.

Overnight Completion

The overnight interval (00:00-07:59) showed an increase from two of 11 patients (18.2%) in cycle 1 to four of 10 patients (40.0%) in cycle 2. The absolute increase was 21.8 percentage points, but the small denominators mean that each individual record materially changes the percentage. The pattern is compatible with improvement but should not be over-interpreted. It also indicates that the overnight process remained vulnerable: six of 10 records in the second cycle did not show a completed form. Further work should distinguish between avoidable omissions and appropriate deferral, such as when a patient is asleep or unable to engage, and should ensure that deferred assessments are revisited when clinically appropriate.

What the Project Can and Cannot Establish

These data demonstrate a difference in recorded completion between two audit periods. They do not establish that the intervention caused the difference, because there was no concurrent control group and the available material does not document all changes in staffing, workload, patient characteristics, or local practice. Nor can the project determine whether more complete AMTS documentation improved recognition of cognitive impairment, delirium care, length of stay, or other patient outcomes. The most defensible conclusion is that the completion measure improved after a multicomponent intervention and that the result warrants further testing and monitoring.

Measurement Considerations for Future Cycles

A stronger measurement plan would define the denominator in advance, specify whether patients who are unable to participate remain in the denominator, and record a reason when AMTS is not completed. This would reduce ambiguity in interpreting missing forms. The same audit windows and inclusion criteria should be used in each cycle. If data collection permits, the team could report the number eligible, the number assessed, the number with a documented reason for deferral, and the number with no recorded explanation. Reporting counts alongside percentages is especially important for small time intervals.

Where feasible, a run chart using weekly or otherwise regularly spaced observations would show whether the process changed over time and whether the change was sustained. Two aggregate points are insufficient to distinguish a special-cause change from normal variation. A future analysis could stratify completion by day, evening, and night shifts, and examine whether performance differs by ward area or clinician group if these data are collected consistently. Any statistical analysis should be selected after the data structure, and denominators are verified, rather than added retrospectively to imply certainty.

Balancing Measures and Patient-Centred Exceptions

Improvement efforts should avoid creating pressure to complete a cognitive test when it is not clinically appropriate or when the patient cannot meaningfully participate. A balancing measure could record whether staff report that the new process adds unreasonable time or duplicates existing documentation. Another useful measure would be the proportion of initially deferred assessments that are revisited later, if that is part of the agreed local pathway. These measures would help the team distinguish a genuinely more reliable process from a rise in form completion that does not reflect appropriate clinical practice.

Sustainability and spread

Sustainability is likely to depend on how the task is embedded in routine work. Posters and one-off teaching sessions may have a diminishing effect as staff rotate or new clinicians join the service. Possible next steps include adding the expectation to induction, agreeing a named role responsible for checking completion, and reviewing a small sample of records at regular team meetings. If a prompt is added to an electronic or paper clerking template, the team should test whether it supports completion without increasing alert fatigue or encouraging superficial documentation. Any spread to other departments should follow local testing and adaptation rather than assuming that the same intervention will work in every setting.

Strengths

The project addressed a practical documentation issue in a real acute medical setting and included two audit cycles rather than relying on a single baseline measurement. Staff feedback helped the team consider reasons for incomplete documentation, and the SEIPS framework provided a structured way to relate those barriers to the work system. The intervention package used accessible approaches, including teaching, posters, awareness activities, and team discussion, that could be delivered without a complex technical implementation. The second cycle also allowed the team to compare completion against a stated local target.

Limitations

The project has several limitations beyond those inherent to a small, single-centre QIP. The calendar year and exact dates of the March and June cycles remain to be confirmed in the manuscript. Full survey methods, respondent numbers, response frequencies, and the wording of survey questions were not available in the source material reviewed for this draft. The intervention package included several elements introduced together, preventing attribution of the observed change to any one component. The sample sizes were modest, particularly within individual time intervals. The available data measured documentation rather than clinical appropriateness, assessment quality, downstream action, or patient outcomes. Finally, the governance determination has not yet been confirmed and must be resolved before submission.

Implications for practice

For the local team, the results provide a reason to continue improving the reliability of AMTS documentation while avoiding the assumption that a single educational intervention will solve all omissions. The most useful next step is likely to be a focused third cycle that targets the remaining gap, particularly overnight, and tests a small number of clearly defined changes. The clinical team should decide whether AMTS is the correct locally required tool for the intended population and how to document clinically appropriate deferral. The results should be shared with the staff who complete the task so that any new workflow is feasible and understood.

Future work

Future work should prioritise verification and sustainability. First, the team should preserve a clear audit dataset with dates, inclusion criteria, time interval, completion status, and reason for non-completion. Second, the team should decide whether the 60% target remains appropriate or whether a more ambitious target is justified by local policy and clinical workflow. Third, a third PDSA cycle could test a single workflow-linked prompt or explicit ownership arrangement and assess its effect over several weeks. Fourth, the team should repeat staff feedback after implementation to see whether barriers have changed. Finally, follow-up measurement should assess whether the change persists after staff turnover and whether documentation is accompanied by appropriate clinical action when a screen is abnormal.

## Conclusions

After a SEIPS-informed package of education and awareness measures, recorded AMTS completion increased from 46.7% in March to 69.0% in June, using the cycle counts confirmed by the project lead. The June result exceeded the 60% target. Completion during the overnight interval also increased but remained incomplete. These local findings support continued examination of the people, tasks, tools, organisational arrangements, and environmental conditions that influence documentation.

The results are encouraging but do not establish that the intervention alone caused the improvement or that better documentation translated into better patient outcomes. Verification of the second-cycle raw data, clear reporting of local governance, and further cycles focused on reliable workflow prompts, ownership, night-shift barriers, and sustained measurement are needed before submission and wider implementation.
